# Supplementary material for: A central CRMP complex essential for invasion in Toxoplasma gondii
Source: PLoS Biol. 2023 Jan 5;21(1):e3001937. doi: 10.1371/journal.pbio.3001937 (PMC9815656; doi:10.1371/journal.pbio.3001937)
Supplement: S2 Table — (DOCX) [file pbio.3001937.s007.docx]

**Supplementary table S2**

List of gRNAs

| **Name** | **GeneID** | **gRNA** | **location** |
| --- | --- | --- | --- |
| CRMPA | TGGT1_261080 | **G**TCCTGCTTACAGACCGTCGT | C-term |
| CRMPA | TGGT1_261080 | GAGCTCTTCGGCCTCACGGC | intron |
| CRMPA | TGGT1_261080 | **G**CGGCTTGCTCGAGGAAGCAG | extracellular tag |
| CRMPB | TGGT1_292020 | **G**TCTCTAAGCTACTCCTGTTT | C-term |
| CRMPB | TGGT1_292020 | **G**CAGCTAGTCTCGAGTTGTTC | 1089 bp before ATG |
| CRMPB | TGGT1_292020 | **G**TTGCGTAGACAATTGGCGAA | intron |
| CRMPB | TGGT1_292020 | GCGCGTTACAGATGCGATCG | extracellular tag |
| MIC15 | TGGT1_247195 | GCTGGACACGTCCACCCAAG | C-term |
| MIC15 | TGGT1_247195 | **G**CAACAGATACGTTCATTTAC | intron |
| TSP1 | TGGT1_277910 | **G**CCGTGACACCCGAATCCTTT | C-term |
| TSP1 | TGGT1_277910 | GAAGAAGAACGGAAAATCTC | pre ATG |
| TRP | TGGT1_247000 | **G**TCTCCTTCACTGACTTGCTG | C-term |
| TRP | TGGT1_247000 | **G**CGTCACAAATCGAACAGCCA | pre ATG |
| hypothetical | TGGT1_285500 | **G**CATGTGATGATTGTGAAAAG | C-term |
| hypothetical | TGGT1_285500 | **G**TTCTGGATAGCCCATTCGCG | pre ATG |
| RON2 | TGGT1_300100 | **G**AAACGTCCGAAAAACAGACG | C-term |
| MIC8 | TGGT1_245490 | **G**TTGGCCTTCATTCTGTCTTG | pre ATG |
| MIC8 | TGGT1_245490 | **G**TCTGTCTGATCAGTGCTTTC | internal after EGF |

Sequence of tags

3xHA

GCTAAAATTGGAAGTGGAGGATACCCGTACGACGTCCCGGACTACGCTGGCTATCCCTATGATGTGCCCGATTATGCGTATCCTTACGATGTTCCAGATTATGCCTAAATAACTTCGTATAGCATACATTATACGAAGTTAT

sYFP2

GCTAAAATTGGAAGTGGAGGACGGATGGTGAGCAAGGGCGAGGAGCTGTTCACCGGGGTGGTGCCCATCCTGGTCGAGCTGGACGGCGACGTAAACGGCCACAAGTTCAGCGTGTCCGGCGAGGGCGAGGGCGATGCCACCTACGGCAAGCTGACCCTGAAGCTGATCTGCACCACCGGCAAGCTGCCCGTGCCCTGGCCCACCCTCGTGACCACCCTGGGCTACGGCGTGCAGTGCTTCGCCCGCTACCCCGACCACATGAAGCAGCACGACTTCTTCAAGTCCGCCATGCCCGAAGGCTACGTCCAGGAGCGCACCATCTTCTTCAAGGACGACGGCAACTACAAGACCCGCGCCGAGGTGAAGTTCGAGGGCGACACCCTGGTGAACCGCATCGAGCTGAAGGGCATCGACTTCAAGGAGGACGGCAACATCCTGGGGCACAAGCTGGAGTACAACTACAACAGCCACAACGTCTATATCACCGCCGACAAGCAGAAGAACGGCATCAAGGCCAACTTCAAGATCCGCCACAACATCGAGGACGGCGGCGTGCAGCTCGCCGACCACTACCAGCAGAACACCCCCATCGGCGACGGCCCCGTGCTGCTGCCCGACAACCACTACCTGAGCTACCAGTCCAAGCTGAGCAAAGACCCCAACGAGAAGCGCGATCACATGGTCCTGCTGGAGTTCGTGACCGCCGCCGGGATCACTCTCGGCATGGACGAGCTGTACAAGTAGATAACTTCGTATAGCATACATTATACGAAGTTAT

HALO

GCTAAAATTGGAAGTGGAGGACGGCCTAGGCTCGAGCCAACCACTGAGGATCTGTACTTTCAGAGCGATAACGATGGATCCGAAATCGGTACTGGCTTTCCATTCGACCCCCATTATGTGGAAGTCCTGGGCGAGCGCATGCACTACGTCGATGTTGGTCCGCGCGATGGCACCCCTGTGCTGTTCCTGCACGGTAACCCGACCTCCTCCTACGTGTGGCGCAACATCATCCCGCATGTTGCACCGACCCATCGCTGCATTGCTCCAGACCTGATCGGTATGGGCAAATCCGACAAACCAGACCTGGGTTATTTCTTCGACGACCACGTCCGCTTCATGGATGCCTTCATCGAAGCCCTGGGTCTGGAAGAGGTCGTCCTGGTCATTCACGACTGGGGCTCCGCTCTGGGTTTCCACTGGGCCAAGCGCAATCCAGAGCGCGTCAAAGGTATTGCATTTATGGAGTTCATCCGCCCTATCCCGACCTGGGACGAATGGCCAGAATTTGCCCGCGAGACCTTCCAGGCCTTCCGCACCACCGACGTCGGCCGCAAGCTGATCATCGATCAGAACGTTTTTATCGAGGGTACGCTGCCGATGGGTGTCGTCCGCCCGCTGACTGAAGTCGAGATGGACCATTACCGCGAGCCGTTCCTGAATCCTGTTGACCGCGAGCCACTGTGGCGCTTCCCAAACGAGCTGCCAATCGCCGGTGAGCCAGCGAACATCGTCGCGCTGGTCGAAGAATACATGGACTGGCTGCACCAGTCCCCTGTCCCGAAGCTGCTGTTCTGGGGCACCCCAGGCGTTCTGATCCCACCGGCCGAAGCCGCTCGCCTGGCCAAAAGCCTGCCTAACTGCAAGGCTGTGGACATCGGCCCGGGTCTGAATCTGCTGCAAGAAGACAACCCGGACCTGATCGGCAGCGAGATCGCGCGCTGGCTGTCTACTCTGGAGATTTCCGGTTAAATAACTTCGTATAGCATACATTATACGAAGTTATAGATC

SNAP

GCTAAAATTGGAAGTGGAGGACGGGAATTCATGGATAAGGACTGCGAAATGAAGCGCACCACCCTGGATAGCCCTCTGGGCAAGCTGGAACTGTCTGGGTGCGAACAGGGCCTGCACGAGATCAAGCTGCTGGGCAAAGGAACATCTGCCGCCGACGCCGTGGAAGTGCCTGCCCCAGCCGCCGTGCTGGGCGGACCAGAGCCACTGATGCAGGCCACCGCCTGGCTCAACGCCTACTTTCACCAGCCTGAGGCCATCGAGGAGTTCCCTGTGCCAGCCCTGCACCACCCAGTGTTCCAGCAGGAGAGCTTTACCCGCCAGGTGCTGTGGAAACTGCTGAAAGTGGTGAAGTTCGGAGAGGTCATCAGCTACCAGCAGCTGGCCGCCCTGGCCGGCAATCCCGCCGCCACCGCCGCCGTGAAAACCGCCCTGAGCGGAAATCCCGTGCCCATTCTGATCCCCTGCCACCGGGTGGTGTCTAGCTCTGGCGCCGTGGGGGGCTACGAGGGCGGGCTCGCCGTGAAAGAGTGGCTGCTGGCCCACGAGGGCCACAGACTGGGCAAGCCTGGATTAGGATAGATAACTTCGTATAGCATACATTATACGAAGTTAT

TurboID

GCTAAAATTGGAAGTGGAGGACGGGAATTCGCTAGCAAAGACAATACTGTGCCTCTGAAGCTGATCGCTCTCCTGGCTAATGGCGAGTTCCATAGTGGCGAACAGCTGGGAGAAACCCTGGGCATGTCCAGGGCCGCTATCAACAAGCACATTCAGACTCTGCGCGACTGGGGCGTGGACGTGTTCACCGTGCCCGGAAAGGGCTACTCTCTGCCCGAGCCTATCCCGCTGCTGAACGCTAAACAGATTCTGGGACAGCTGGACGGCGGGAGCGTGGCAGTCCTGCCTGTGGTCGACTCCACCAATCAGTACCTGCTGGATCGAATCGGCGAGCTGAAGAGTGGGGATGCTTGCATTGCAGAATATCAGCAGGCAGGGAGAGGAAGCAGAGGGAGGAAATGGTTCTCTCCTTTTGGAGCTAACCTGTACCTGAGTATGTTTTGGCGCCTGAAGCGGGGACCAGCAGCAATCGGCCTGGGCCCGGTCATCGGAATTGTCATGGCAGAAGCGCTGCGAAAGCTGGGAGCAGACAAGGTGCGAGTCAAATGGCCCAATGACCTGTATCTGCAGGATAGAAAGCTGGCAGGCATCCTGGTGGAGCTGGCCGGAATAACAGGCGATGCTGCACAGATCGTCATTGGCGCCGGGATTAACGTGGCTATGAGGCGCGTGGAGGAAAGCGTGGTCAATCAGGGCTGGATCACACTGCAGGAAGCAGGGATTAACCTGGACAGGAATACTCTGGCCGCTACGCTGATCCGAGAGCTGCGGGCAGCCCTGGAACTGTTCGAGCAGGAAGGCCTGGCTCCATATCTGCCACGGTGGGAGAAGCTGGATAACTTCATCAATAGACCCGTGAAGCTGATCATTGGGGACAAAGAGATTTTCGGGATTAGCCGGGGGATTGATAAACAGGGAGCCCTGCTGCTGGAACAGGACGGAGTTATCAAACCCTGGATGGGCGGAGAAATCAGTCTGCGGTCTGCCGAAAAGCTGCAGTAGATAACTTCGTATAGCATACATTATACGAAGTTAT

List of parasites strains generated in RH-DiCre

RH-LoxP-CRMPB-3HA^LoxP^

RH-MIC8extrac.^floxed^

RH-CRMPB-3HA^floxed^

RH-CRMPB-3HA^floxed^ CRMPA-sYFP2

RH-CRMPA-3HA^floxed^

RH-CRMPA-3HA^floxed^ CRMPB-sYFP2

RH-SNAP-CRMPB-3HA^floxed^ CRMPA-sYFP2

RH-Halo-CRMPA-3HA^floxed^ CRMPB-sYFP2

RH-sYFP2-CRMPB-3HA^floxed^ CRMPA-sYFP2

RH-CRMPB-3HA^floxed^ CRMPA-sYFP2 RON2-SNAP

RH-CRMPA-3HA^floxed^ GCC2-sYFP2 RON2-SNAP

RH- CRMPB 3HA^floxed^ MIC15-Halo

RH- CRMPB 3HA^floxed^ TSP1-Halo

RH- CRMPB 3HA^floxed^ 247000-Halo

RH- CRMPB 3HA^floxed^ 285500-Halo

RH- CRMPB- TurboID

RH- CRMPB-TurboID CRMPA 3HA^floxed^

RH- CRMPA–TurboID

RH- CRMPA-TurboID CRMPB 3HA^floxed^

RH -MIC15-3HA^floxed^

RH -TSP1-3HA^floxed^

RH -TSP1-3HA^floxed^

RH-CRMPB-Halo CRMPA-3HA^floxed^

Input data for structure prediction in Figure 6E

TgCRMPB_CRMPA_TMs_runs5recycle3

HPCVDDVRCRGGSHNSCVHGSAGYLCSACEEDYSEIRYKSGCQACLPLWLDSFIFILMRLVVCGIIWIITALTIIAVQQQACIHPVLIRIVMSHMFFLSVYGLMPATSQSQLAGWASIYRLFFFEFYFALHPYFKMPCFFRSLGIVMPEAHVWYWQHFFQIFVPFIDAVLLTIIGAICVATYKVMYSAYISRVLVVLEEARQAHGDDMWTEKTIRKIESERCLGMFRYIYGTSTPWENFVRLCTDLIPAYTAIWFWHFPTFVIECTMLMGCIETRYKSEDPISVLAAFPVQICSFENPYFLSGLILGGVGLLVWGVGSIAGFVAYMSGDHSSDTIEQRFKHGFLVNGYQYAYRWWEGVIGLRKTCVALIITMYVHANASGAQEIFRNSANLALTVLSTALQLQLEPFDKRSHDMANRMEFYGLMVNIIIGVIFQGSYYFEVFKYMGAIPLAVAIFYYLYVLWSLFVEWGRMVMMRPHLVS:TECPIQNACLSHGVCSETMTEYLCSECRRGYTNTFSKGEICTSCPSMVWNIVCLMGYYLATLLFNIVMTYMNVAAGFNRRSIHSIVIKIASNYLTGISVLSVIDFSTIAFPSWITDLTATVTETVSAKHSTRLMSVDCLLRDNFDLSFSESFFYTMVFYALIPIALPIVATIIMSIIVYRVRAWYHNSTQRKLELLKQTMQYGLYSLAQQLKEKYEEDRVFMIFRYIALPGESIFRRAAKFMEDMIPIYVTVLFFVYSSTTRNMLSLLDCTYIDFGRAHQAKYFLRAAMSVECTDILSGPYFKFFAVGITGLLVWSIGIPLSCFLVLYVNRKTLNSRETRLKYGFLHNGFVKKYWYWEMVVFARKFLVIVVSSVALIPSADKNGSRVWLAVVIAVIFLIIHLVTQPFDKRSYLTLDKLENHSMTIWTITLIVLAMMIGSDFSGSVNMALLLFVAVLSCMFILEVGVSLMFAYFDNVRTQQTFFRVPVIG

Input data for structure prediction in Figure S7A

TgCRMPA_CRMPA_TMs

TECPIQNACLSHGVCSETMTEYLCSECRRGYTNTFSKGEICTSCPSMVWNIVCLMGYYLATLLFNIVMTYMNVAAGFNRRSIHSIVIKIASNYLTGISVLSVIDFSTIAFPSWITDLTATVTETVSAKHSTRLMSVDCLLRDNFDLSFSESFFYTMVFYALIPIALPIVATIIMSIIVYRVRAWYHNSTQRKLELLKQTMQYGLYSLAQQLKEKYEEDRVFMIFRYIALPGESIFRRAAKFMEDMIPIYVTVLFFVYSSTTRNMLSLLDCTYIDFGRAHQAKYFLRAAMSVECTDILSGPYFKFFAVGITGLLVWSIGIPLSCFLVLYVNRKTLNSRETRLKYGFLHNGFVKKYWYWEMVVFARKFLVIVVSSVALIPSADKNGSRVWLAVVIAVIFLIIHLVTQPFDKRSYLTLDKLENHSMTIWTITLIVLAMMIGSDFSGSVNMALLLFVAVLSCMFILEVGVSLMFAYFDNVRTQQTFFRVPVIG

:TECPIQNACLSHGVCSETMTEYLCSECRRGYTNTFSKGEICTSCPSMVWNIVCLMGYYLATLLFNIVMTYMNVAAGFNRRSIHSIVIKIASNYLTGISVLSVIDFSTIAFPSWITDLTATVTETVSAKHSTRLMSVDCLLRDNFDLSFSESFFYTMVFYALIPIALPIVATIIMSIIVYRVRAWYHNSTQRKLELLKQTMQYGLYSLAQQLKEKYEEDRVFMIFRYIALPGESIFRRAAKFMEDMIPIYVTVLFFVYSSTTRNMLSLLDCTYIDFGRAHQAKYFLRAAMSVECTDILSGPYFKFFAVGITGLLVWSIGIPLSCFLVLYVNRKTLNSRETRLKYGFLHNGFVKKYWYWEMVVFARKFLVIVVSSVALIPSADKNGSRVWLAVVIAVIFLIIHLVTQPFDKRSYLTLDKLENHSMTIWTITLIVLAMMIGSDFSGSVNMALLLFVAVLSCMFILEVGVSLMFAYFDNVRTQQTFFRVPVIG

TgCRMPB_CRMPB_TMs

HPCVDDVRCRGGSHNSCVHGSAGYLCSACEEDYSEIRYKSGCQACLPLWLDSFIFILMRLVVCGIIWIITALTIIAVQQQACIHPVLIRIVMSHMFFLSVYGLMPATSQSQLAGWASIYRLFFFEFYFALHPYFKMPCFFRSLGIVMPEAHVWYWQHFFQIFVPFIDAVLLTIIGAICVATYKVMYSAYISRVLVVLEEARQAHGDDMWTEKTIRKIESERCLGMFRYIYGTSTPWENFVRLCTDLIPAYTAIWFWHFPTFVIECTMLMGCIETRYKSEDPISVLAAFPVQICSFENPYFLSGLILGGVGLLVWGVGSIAGFVAYMSGDHSSDTIEQRFKHGFLVNGYQYAYRWWEGVIGLRKTCVALIITMYVHANASGAQEIFRNSANLALTVLSTALQLQLEPFDKRSHDMANRMEFYGLMVNIIIGVIFQGSYYFEVFKYMGAIPLAVAIFYYLYVLWSLFVEWGRMVMMRPHLVS: HPCVDDVRCRGGSHNSCVHGSAGYLCSACEEDYSEIRYKSGCQACLPLWLDSFIFILMRLVVCGIIWIITALTIIAVQQQACIHPVLIRIVMSHMFFLSVYGLMPATSQSQLAGWASIYRLFFFEFYFALHPYFKMPCFFRSLGIVMPEAHVWYWQHFFQIFVPFIDAVLLTIIGAICVATYKVMYSAYISRVLVVLEEARQAHGDDMWTEKTIRKIESERCLGMFRYIYGTSTPWENFVRLCTDLIPAYTAIWFWHFPTFVIECTMLMGCIETRYKSEDPISVLAAFPVQICSFENPYFLSGLILGGVGLLVWGVGSIAGFVAYMSGDHSSDTIEQRFKHGFLVNGYQYAYRWWEGVIGLRKTCVALIITMYVHANASGAQEIFRNSANLALTVLSTALQLQLEPFDKRSHDMANRMEFYGLMVNIIIGVIFQGSYYFEVFKYMGAIPLAVAIFYYLYVLWSLFVEWGRMVMMRPHLVS

Input data for structure prediction in Figure S7B

PbCRMP1_CRMP3_TMs

IKCPISGACLGKNNCHNTMDNYLCIECKKGYTNSFTKSKCVRCPNNIANIILLLLIYIIFCFIIIIISYLNISSGFYRRSIHSIIIKIAVNYVSSMLIVNILEDTYLNLPPYAYDVYNKMTNILTSENQKKKIISIDCLLRYYFNLTYNDSFFYTSLFFFLIPIFLMLTLTVILFVILKIYTIIQKEGINNKLNLLGMAKKKNISFLVNSLEANYKKERFIMILRYIKLPDNTMSDSILTFFEDMIPIYATFLFIIHAKTSLRMLQLFDCSYIRYTKKISKYILNSSSSVQCNFKTNDYLKFFILGISGTVLWVLGIPFLAFFVLYNNRHNLFHENIRIKYGFLHNGYLPNRWYWEVVVFIRKIIVLFVTTVIVFPSNKEKIYKLLIITFVSIFSLCIHFIFQPFDKRNFFILNKLENFSLYIWVFTIMIISVLMHVNLNEFINLLVFFFIILLHTIFFIKLLICLFYECISNLRATF:HICPIMKKCVGGINNLCYEGSEGYLCSNCSKKYDTKYFRSQCFKCQKTKIEIFNFILFKAVFYALIFFLIYLNYFCCIKKNFVFIGIFKIWYAFIISFLPYIFIMDSNSPREENYILYSQFFTSLPIKFITQHLKINCFINSYNNEKYIYVWYVQRFIKIAEPIIDCFFLVCIFICFYIIYTWWNYQKISLIQKVIKKQINKEYNKEYYKYVSDFYYHYYQKGIIDTINNKRNKTYLEYMKYDCDYFSSYNSLSFRTPLLKKWKNKKCSKKLDKSKLHSVTSWKSNKNQSSKWINQKVSNKSHKWINKKEKKQKNKTISLDDINFLESSSFSRNKELTKENSKEFDFLDVINFKKDISHKIKKTSSLPIEENKTKDNYWTSICLLNIYNIKAMGIFRYIHPPNISIHKKIGSILSDLNAIYIIVFYIHFPFTLMSILELIWCQSTKYKNKLPILILYHMPSQVCSFQNKLFLSGVLFSGFFFFIYLFLFIKYFYGTFKNFKVFQSYDRDFKCYFLFNGYNYQNRCWDFVNIIKIVLFVTSFMCQFYTNKINNSKYFIFSCIIFIIITEITLILLYSPYDKRSNNVLQKLSLLSIFSVLITYLTTHFSFFFDFYIISALPFILFIYFHIHTINKIVLEFALYKNILMKPTR

PbCRMP1_CRMP4_TMs

IKCPISGACLGKNNCHNTMDNYLCIECKKGYTNSFTKSKCVRCPNNIANIILLLLIYIIFCFIIIIISYLNISSGFYRRSIHSIIIKIAVNYVSSMLIVNILEDTYLNLPPYAYDVYNKMTNILTSENQKKKIISIDCLLRYYFNLTYNDSFFYTSLFFFLIPIFLMLTLTVILFVILKIYTIIQKEGINNKLNLLGMAKKKNISFLVNSLEANYKKERFIMILRYIKLPDNTMSDSILTFFEDMIPIYATFLFIIHAKTSLRMLQLFDCSYIRYTKKISKYILNSSSSVQCNFKTNDYLKFFILGISGTVLWVLGIPFLAFFVLYNNRHNLFHENIRIKYGFLHNGYLPNRWYWEVVVFIRKIIVLFVTTVIVFPSNKEKIYKLLIITFVSIFSLCIHFIFQPFDKRNFFILNKLENFSLYIWVFTIMIISVLMHVNLNEFINLLVFFFIILLHTIFFIKLLICLFYECISNLRATF:HPCNLPDRCLGTITNLCYEGSTGYQCNSCSKNYDMKYFKSKCTKCRNIYHEILSIILLKIIYYVIIIYIVSLNYNSCLNNLYVSGVLFRIWLNCSFSFISLGFFSPNNLSFITRYWYVYKEIFLYHLNFCSPYIRVGCFMSYYNTDITYKNIWYIQKYLNIFTFFFDVFFITLILFIIMKITNFWHRKKIQNFEIMLSTVPEVYDKFKIEQNDKKKIKNKKTPKIHILNSNHEDQEDGSQNNLTLIHDLEKNESQSDEYATHGDAQNFQNNRNDCNSQDEQGEEPHTLKNNTNVSVEKKINFLTKNKKSMLNNSQIENKLTEENENIHDENYELKYIKKEHTMYSQFLNEAVEYIYDKKMFGPWRFIHKKNDCFRKRFLGFISDSIPCYILMIIISTPYILLETVQLFYCKSIKFKSEKSELYLAYLNTQKCTTSSASFVMGLIVAFVVLLFYIATLILLLYLYSKRKTIKLFNKFLKNLLSGYRQGKEIFEVIFLLKNIILVVMIAFTIYYQYYYIVLITLTLTLFSILELISDPFDRRSFNILNISLRVGSVLNIFFSIMIWGSFYLNYERHILFPFFIVILYHFYMVHNIVKEVILSKYFITIQTY

PbCRMP2_CRMP4_TMs

VECPIKDACLYNGKCYESMTNFLCGECKKGYTNNFSKLNLCIKCSGIIANILYILFVNMFALLFIVIMAYLNVFTGTNRKSVHSIVIKIGINYFSCMKLFYIIGTSEMYFPINLSSHVNYMTKYIKRLLKVKKNYGIYCILTSFNISHANAYFYGMLYYAFKPILLAMILTILMYIVVEMYKYKVRDKTNIKLRVIDEIKVLGNNKLYDEIMQELISERGLVLFRYISIPGDSRFKRIKIFFEDMIPIHVTLLFFMHTEITYYMLTLLNCKAIYYNDKFIEQYMSFAPSVKCDLSKDYAKFFILGISGLVVWGIGIPLMSYLVLYKNRRKLHNENVLLKYGFLNNGFNFQFWYWETIVFLRKNMILLISTVSLLKTTRVLGTTMWLFTCVSSFFLILQIILQPFDSRNYHILNRLETFSMVAWTISLIILAFLTVSSASSTVNFYVLLSLLFFNCIFMANILIVLCNSYMENLRHIK:HPCNLPDRCLGTITNLCYEGSTGYQCNSCSKNYDMKYFKSKCTKCRNIYHEILSIILLKIIYYVIIIYIVSLNYNSCLNNLYVSGVLFRIWLNCSFSFISLGFFSPNNLSFITRYWYVYKEIFLYHLNFCSPYIRVGCFMSYYNTDITYKNIWYIQKYLNIFTFFFDVFFITLILFIIMKITNFWHRKKIQNFEIMLSTVPEVYDKFKIEQNDKKKIKNKKTPKIHILNSNHEDQEDGSQNNLTLIHDLEKNESQSDEYATHGDAQNFQNNRNDCNSQDEQGEEPHTLKNNTNVSVEKKINFLTKNKKSMLNNSQIENKLTEENENIHDENYELKYIKKEHTMYSQFLNEAVEYIYDKKMFGPWRFIHKKNDCFRKRFLGFISDSIPCYILMIIISTPYILLETVQLFYCKSIKFKSEKSELYLAYLNTQKCTTSSASFVMGLIVAFVVLLFYIATLILLLYLYSKRKTIKLFNKFLKNLLSGYRQGKEIFEVIFLLKNIILVVMIAFTIYYQYYYIVLITLTLTLFSILELISDPFDRRSFNILNISLRVGSVLNIFFSIMIWGSFYLNYERHILFPFFIVILYHFYMVHNIVKEVILSKYFITIQTY

PbCRMP2_CRMP3_TMs

VECPIKDACLYNGKCYESMTNFLCGECKKGYTNNFSKLNLCIKCSGIIANILYILFVNMFALLFIVIMAYLNVFTGTNRKSVHSIVIKIGINYFSCMKLFYIIGTSEMYFPINLSSHVNYMTKYIKRLLKVKKNYGIYCILTSFNISHANAYFYGMLYYAFKPILLAMILTILMYIVVEMYKYKVRDKTNIKLRVIDEIKVLGNNKLYDEIMQELISERGLVLFRYISIPGDSRFKRIKIFFEDMIPIHVTLLFFMHTEITYYMLTLLNCKAIYYNDKFIEQYMSFAPSVKCDLSKDYAKFFILGISGLVVWGIGIPLMSYLVLYKNRRKLHNENVLLKYGFLNNGFNFQFWYWETIVFLRKNMILLISTVSLLKTTRVLGTTMWLFTCVSSFFLILQIILQPFDSRNYHILNRLETFSMVAWTISLIILAFLTVSSASSTVNFYVLLSLLFFNCIFMANILIVLCNSYMENLRHIK:HICPIMKKCVGGINNLCYEGSEGYLCSNCSKKYDTKYFRSQCFKCQKTKIEIFNFILFKAVFYALIFFLIYLNYFCCIKKNFVFIGIFKIWYAFIISFLPYIFIMDSNSPREENYILYSQFFTSLPIKFITQHLKINCFINSYNNEKYIYVWYVQRFIKIAEPIIDCFFLVCIFICFYIIYTWWNYQKISLIQKVIKKQINKEYNKEYYKYVSDFYYHYYQKGIIDTINNKRNKTYLEYMKYDCDYFSSYNSLSFRTPLLKKWKNKKCSKKLDKSKLHSVTSWKSNKNQSSKWINQKVSNKSHKWINKKEKKQKNKTISLDDINFLESSSFSRNKELTKENSKEFDFLDVINFKKDISHKIKKTSSLPIEENKTKDNYWTSICLLNIYNIKAMGIFRYIHPPNISIHKKIGSILSDLNAIYIIVFYIHFPFTLMSILELIWCQSTKYKNKLPILILYHMPSQVCSFQNKLFLSGVLFSGFFFFIYLFLFIKYFYGTFKNFKVFQSYDRDFKCYFLFNGYNYQNRCWDFVNIIKIVLFVTSFMCQFYTNKINNSKYFIFSCIIFIIITEITLILLYSPYDKRSNNVLQKLSLLSIFSVLITYLTTHFSFFFDFYIISALPFILFIYFHIHTINKIVLEFALYKNILMKPTR

Input data for structure prediction in Figure S7C

HPCVDDVRCRGGSHNSCVHGSAGYLCSACEEDYSEIRYKSGCQACLPLWLDSFIFILMRLVVCGIIWIITALTIIAVQQQACIHPVLIRIVMSHMFFLSVYGLMPATSQSQLAGWASIYRLFFFEFYFALHPYFKMPCFFRSLGIVMPEAHVWYWQHFFQIFVPFIDAVLLTIIGAICVATYKVMYSAYISRVLVVLEEARQAHGDDMWTEKTIRKIESERCLGMFRYIYGTSTPWENFVRLCTDLIPAYTAIWFWHFPTFVIECTMLMGCIETRYKSEDPISVLAAFPVQICSFENPYFLSGLILGGVGLLVWGVGSIAGFVAYMSGDHSSDTIEQRFKHGFLVNGYQYAYRWWEGVIGLRKTCVALIITMYVHANASGAQEIFRNSANLALTVLSTALQLQLEPFDKRSHDMANRMEFYGLMVNIIIGVIFQGSYYFEVFKYMGAIPLAVAIFYYLYVLWSLFVEWGRMVMMRPHLVS:TECPIQNACLSHGVCSETMTEYLCSECRRGYTNTFSKGEICTSCPSMVWNIVCLMGYYLATLLFNIVMTYMNVAAGFNRRSIHSIVIKIASNYLTGISVLSVIDFSTIAFPSWITDLTATVTETVSAKHSTRLMSVDCLLRDNFDLSFSESFFYTMVFYALIPIALPIVATIIMSIIVYRVRAWYHNSTQRKLELLKQTMQYGLYSLAQQLKEKYEEDRVFMIFRYIALPGESIFRRAAKFMEDMIPIYVTVLFFVYSSTTRNMLSLLDCTYIDFGRAHQAKYFLRAAMSVECTDILSGPYFKFFAVGITGLLVWSIGIPLSCFLVLYVNRKTLNSRETRLKYGFLHNGFVKKYWYWEMVVFARKFLVIVVSSVALIPSADKNGSRVWLAVVIAVIFLIIHLVTQPFDKR
