# Supplementary figures and images for: A central CRMP complex essential for invasion in Toxoplasma gondii
Source: PLoS Biol. 2023 Jan 5;21(1):e3001937. doi: 10.1371/journal.pbio.3001937 (PMC9815656; doi:10.1371/journal.pbio.3001937)

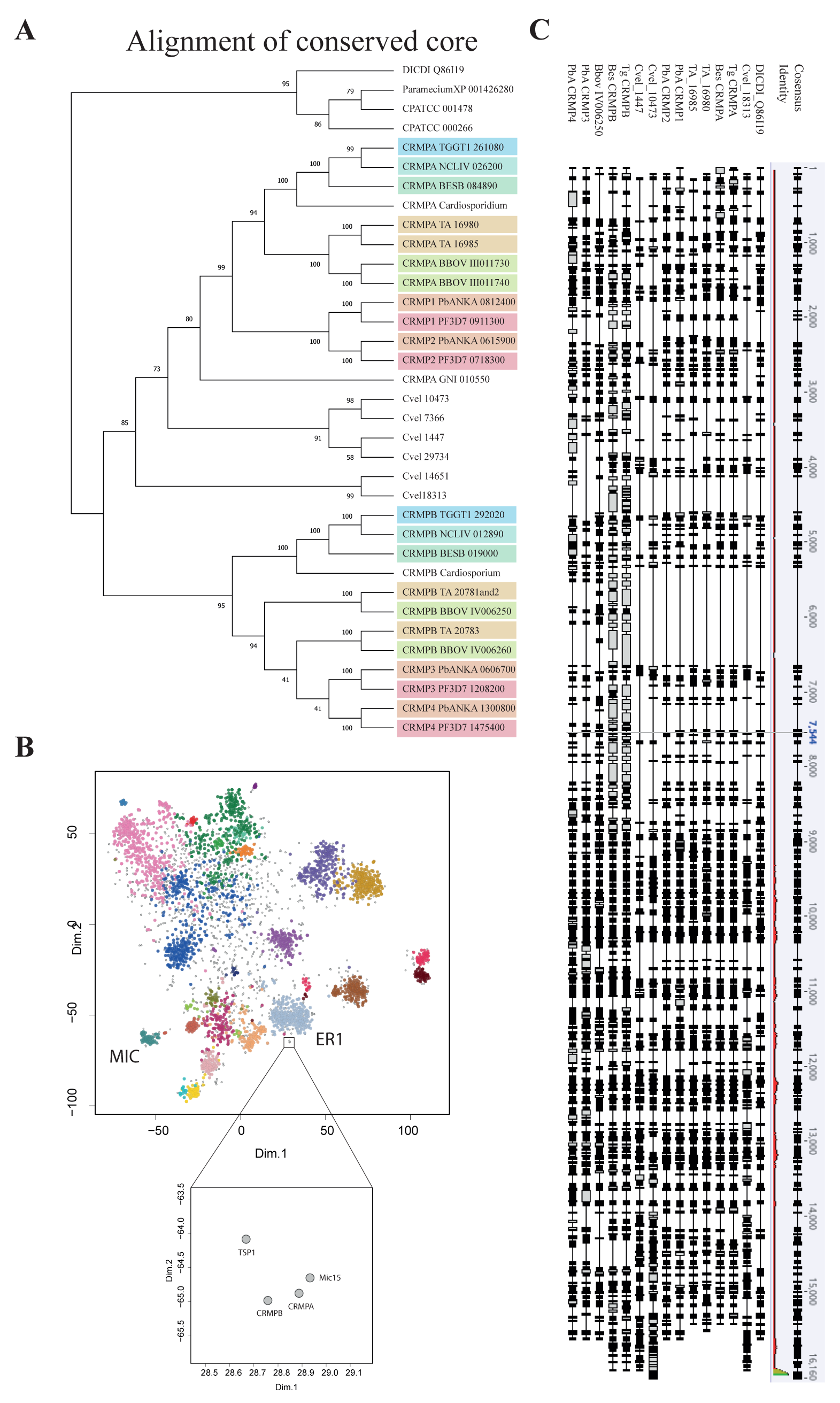

Supplement: S1 Fig — (A) Phylogenetic relationship of CRPMs is shown after alignment of the conserved core region using the PRANK algorithm; bootstrap analysis (100×) supports most branches. Compare with Fig 1A. See also S3 Table. (B) Spatial proteomic map of all T. gondii proteins [30], the micronemal cluster (MIC) and the ER1 cluster are indicated. Insert on the right below the ER cluster shows a separate “minicluster” of CRMPB and CRMPA, grouping together with Thrombospondin typ 1 domain-containing protein (TSP1; TGGT1_277910) and MIC15 (TGGT1_247195). See also https://proteome.shinyapps.io/toxolopittzex/ [30]. (C) A local conservation map and local protein identity and conservation. Insertion are indicated by gray boxes. (TIF) [file pbio.3001937.s009.tif]

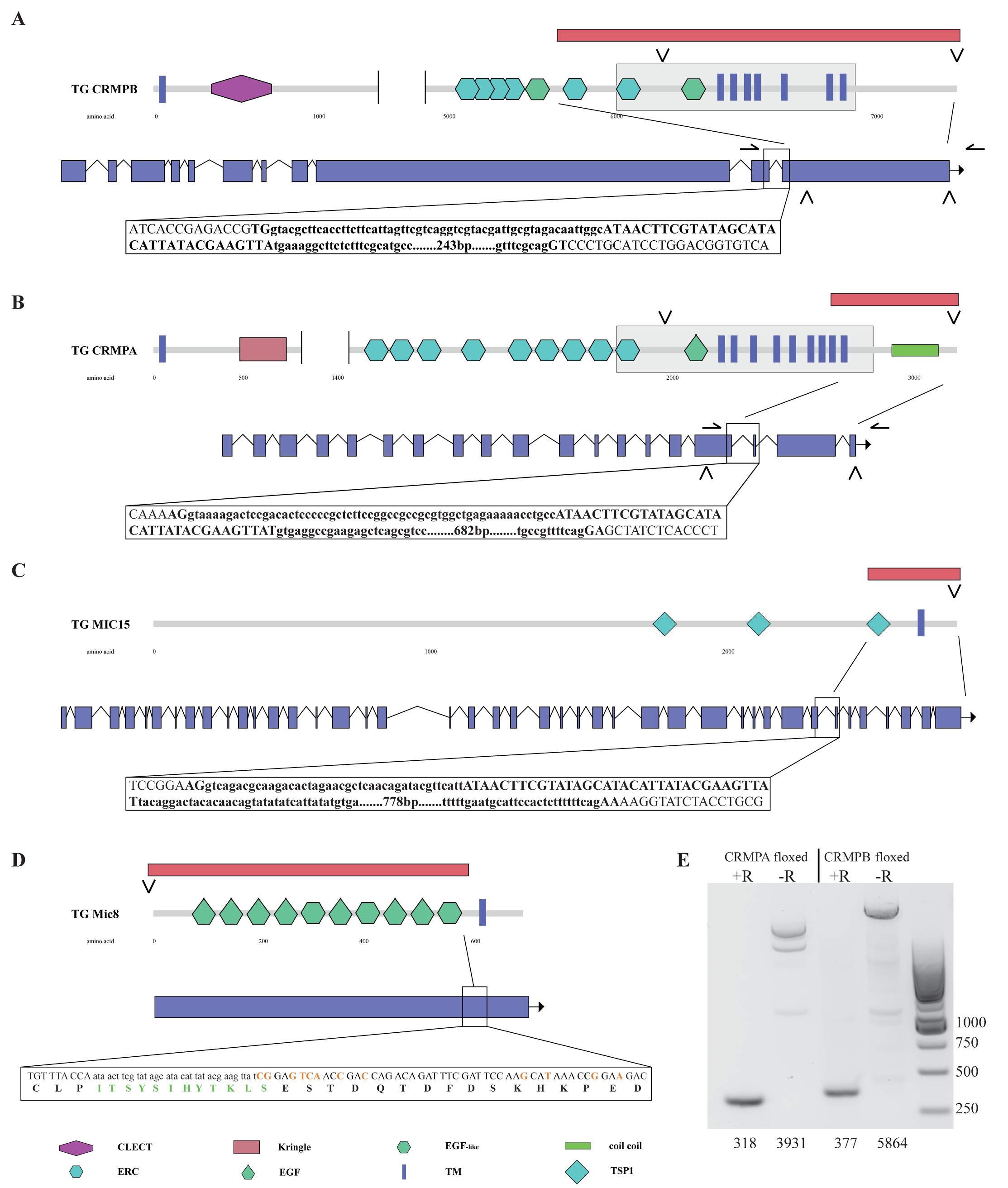

Supplement: S2 Fig — (A) Shown is an overview of the domain architecture and the gene CRMPB of T. gondii. Arrowheads indicate the position of the C-terminal (including the C-terminal LoxP site) and extracellular tag in the gene and the protein model. Position of the LoxP in the intron and the local sequence is shown; relative position within the domain model is indicated by a line and lost sequence after rapamycin induction is indicated by a red bar. (B) Overview of the domain architecture and the gene of CRMPA. Note that only the last transmembrane domain and the C-terminus is lost upon induction with rapamycin. (C) Overview of the domain architecture and the gene MIC15. Position of the LoxP within the intron and the part of the gene lost after rapamycin induction is indicated. (D) Overview of domain architecture of MIC8. The upstream LoxP sequence is 6 bp upstream of the start site (position indicated by an arrow head); the internal LoxP site is 3 bp after the last EGF-like domain inserted as coding as indicated, inserting the protein sequence ITSYSIHYTKLS into the protein (coding sequence in lower case, protein sequence in green). Shielding mutations inserted are indicated with orange letters. Several domains are indicated: CLECT (c-type Lectin or carbohydrate recognition domain), ERC (Ephrin-receptor like), EGF-like (Epidermal growth factor-like), TM (transmembrane domain), Kringle (Kringle domain), EGF (Epidermal growth factor), coil coil (alpha helical coil coil domains), TSP1 (thrombospondin type-1 repeat). (E) Genotyping of RH-CRMPA-3HAfloxed and RH-CRMPB-3HAfloxed after 72 h induction with rapamycin. The respective primers bind outside of the floxed region and are indicated in (A) and (B) with arrows. Estimated sizes are indicated below in basepairs. (TIF) [file pbio.3001937.s010.tif]

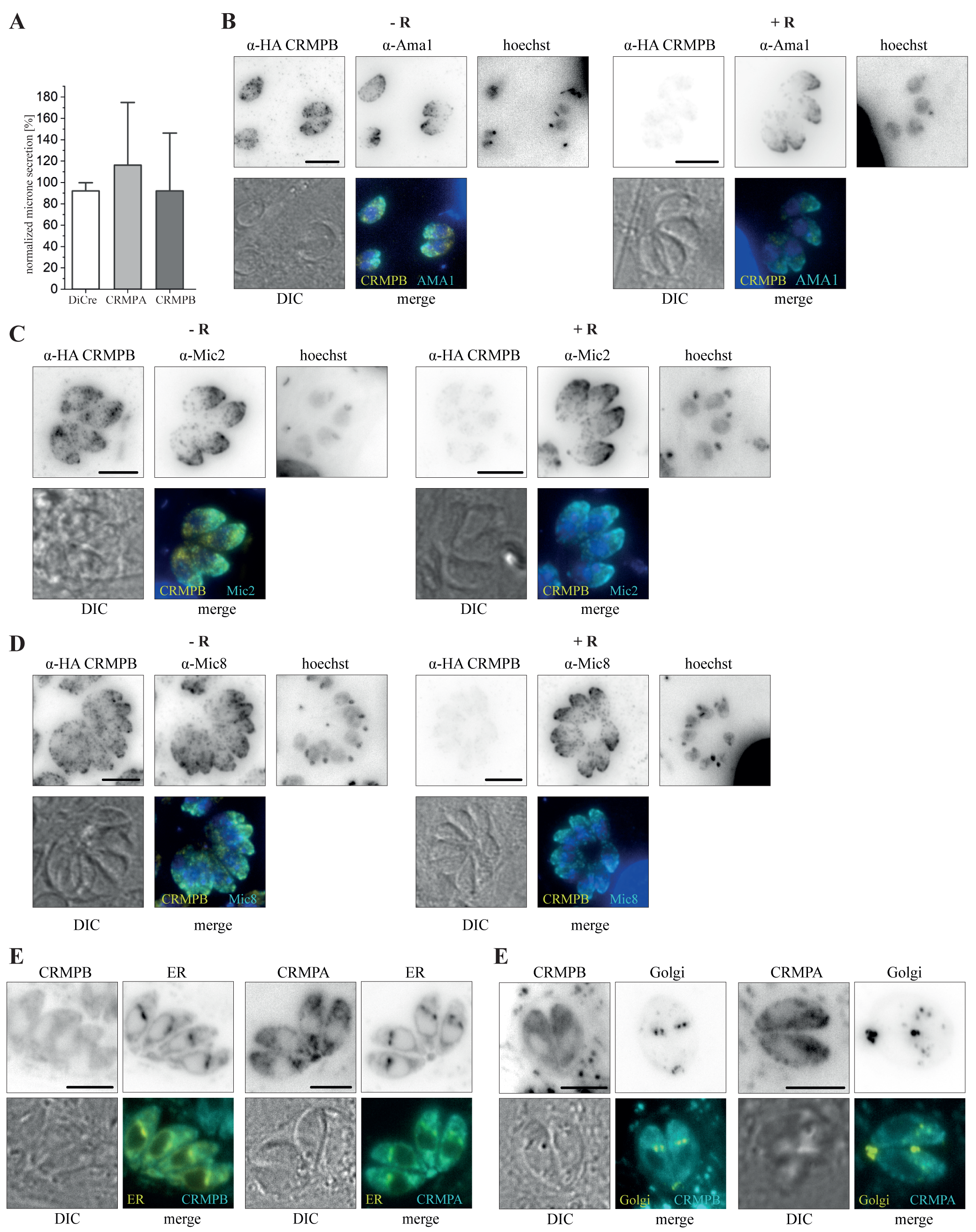

Supplement: S3 Fig — (A) Quantification of microneme secretion measured by secretion of MIC2 relative to GRA1 secretion. DiCre, RH-CRMPA-3HAfloxed, and RH-CRMPB-3HAfloxed were measured 72 h post-rapamycin treatment. See also S1 Data. The abundance and localization of (B) AMA1, (C) MIC2, and (D) MIC8 relative to CRMPB was analyzed in presence and absence (72 h rapamycin) of CRMPB in RH-CRMPB-3HAfloxed. (E) Parasites with extracellular SNAP-tagged CRMPB (RH-SNAP-CRMPB-3HAfloxed CRMPA-sYFP2) or extracellular Halo-tagged CRMPA (RH-Halo-CRMPA-3HAfloxed CRMPB-sYFP2) were transiently transfected with (E) ERD-GFP and (F) GalNac YFP (TGGT1_259530-YFP) [71] and labeled (SNAP-Cell or Halo-Janelia 646) and imaged live 48 h post-transfection. Scale bars are 5 μm. (TIF) [file pbio.3001937.s011.tif]

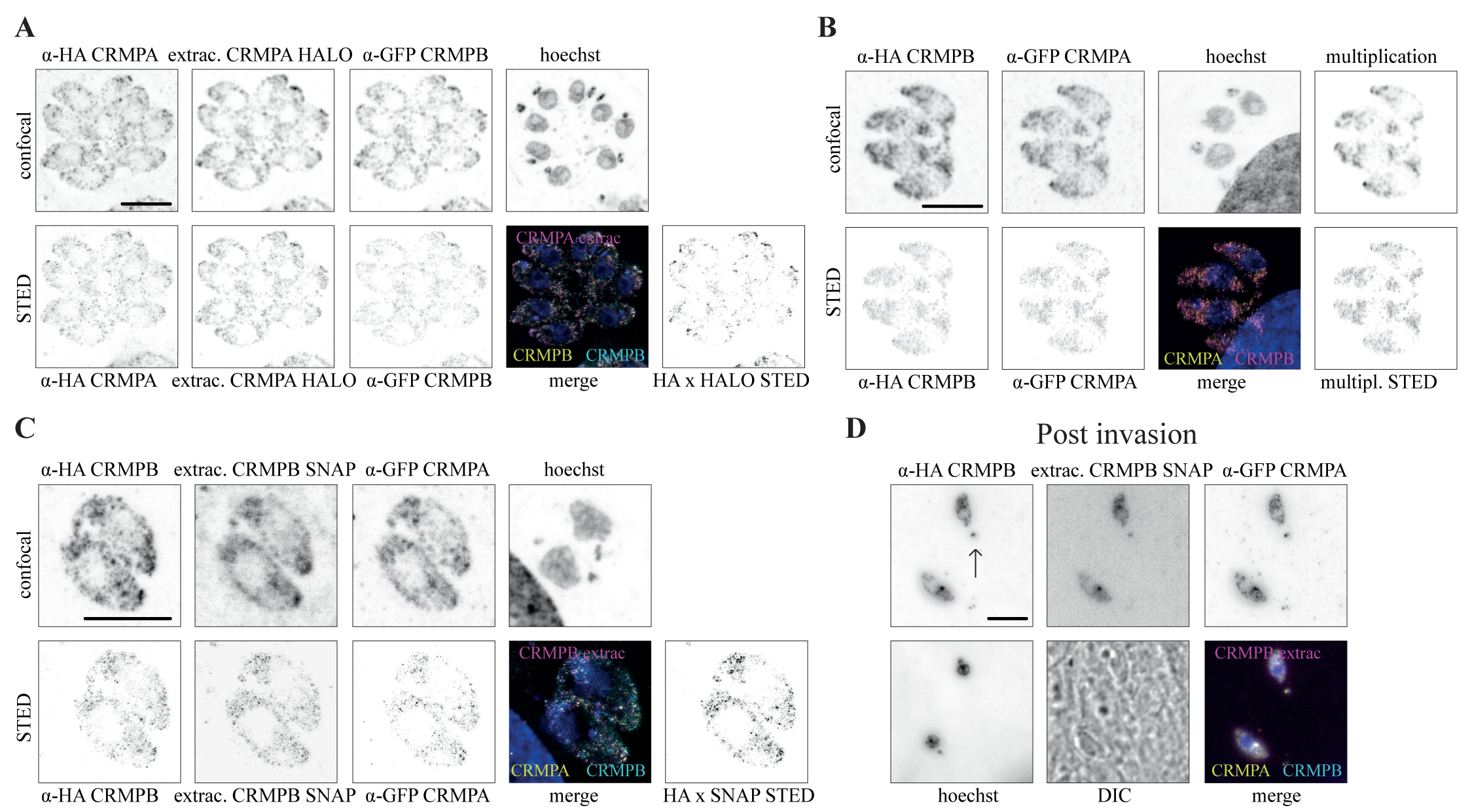

Supplement: S4 Fig — (A) Localization of HALO (Janelia Fluor 646) within the extracellular domain of CRMPA (see S2 Fig) in respect to C-terminally tagged CRMPA (3HA) and C-terminally tagged CRMPB (sYFP2). (B) Localization of RH-CRMPB-3HAfloxed CRMPA-sYFP2 was stained with for HA (Star635P) and sYFP2 (Atto594). Compare with Fig 2A. (C) Localization of SNAP (Sir647) within the extracellular domain of CRMPB (see S2 Fig) in respect to C-terminally tagged CRMPB (3HA) and C-terminally tagged CRMPA (sYFP2). (D) Parasites with extracellular CRMPB SNAP (Janelia 646) after pulse invasion in the presence of proponol. The putative invasion site is marked with an arrow. Scale bars are 5 μm. (TIF) [file pbio.3001937.s012.tif]

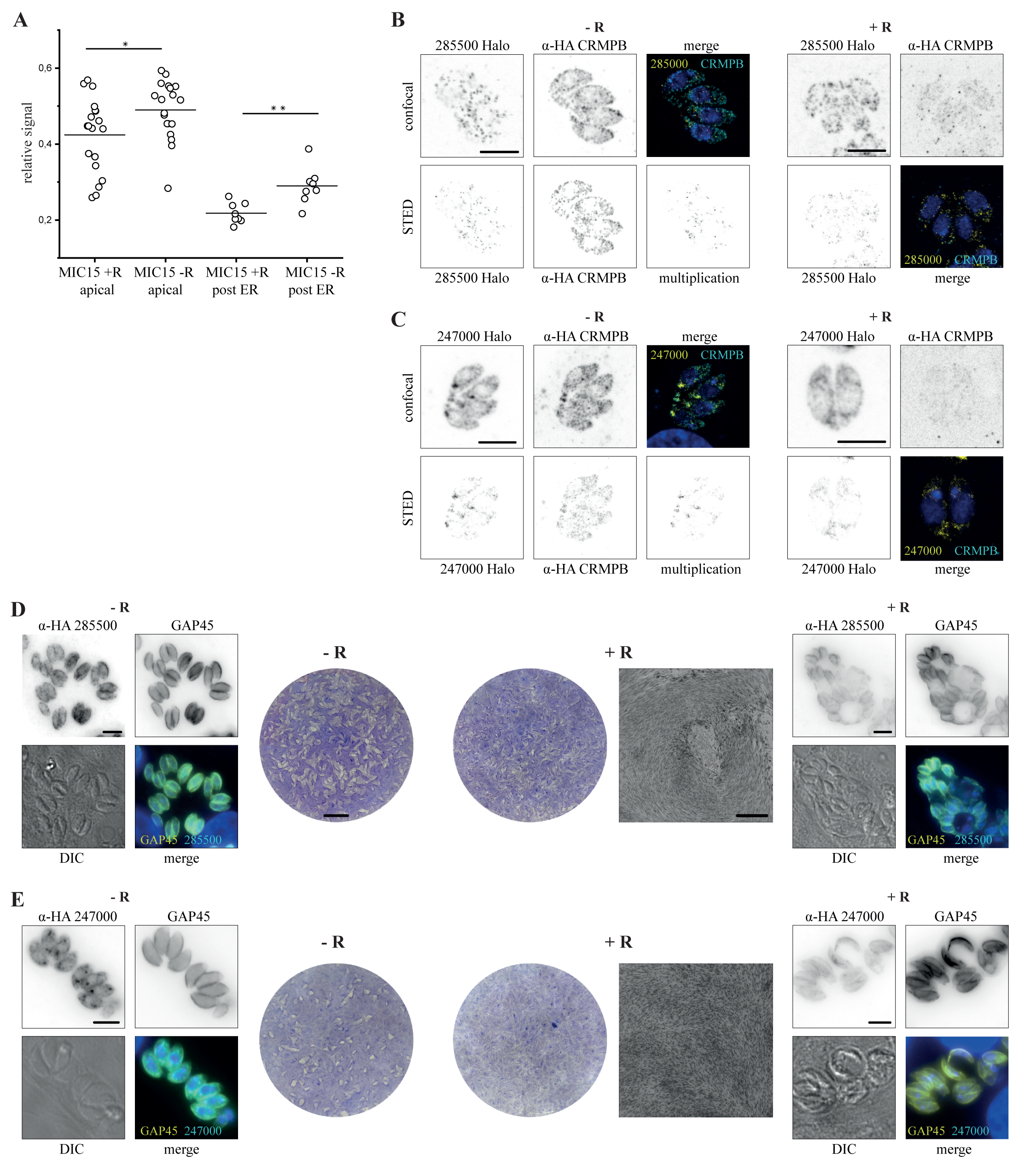

Supplement: S5 Fig — (A) The relative localization of MIC15 was quantified in the presence and absence of CRMPB. Analysis was performed manually (apical of total fluorescence) and automatically (post-ER of total fluorescence). For details, see Methods section and S1 Data. (B) Colocalization of TGGT1_285500 (Halo) with CRMPB and localization of 285500 in the absence of CRMPB (72 h). (C) Colocalization of the tetratricopeptide repeat containing protein (TGGT1_247000) (Halo) with CRMPB and localization of 247000 in the absence of CRMPB (72 h). (D) Localization of 3HA tagged 285500 ±rapamycin (72 h). Plaque assay after 7 days shows a mild growth phenotype. (E) Localization of 3HA tagged 247000 ±rapamycin (72 h). No plaques could be detected 7 days after rapamycin induction. Note that there is some crosstalk with the Gap45 signal in (C) and (D). Scale bars of the plaque assay are 5 mm (scale bar of the higher-resolution area is 500 μm); all other scale bars are 5 μm. (TIF) [file pbio.3001937.s013.tif]

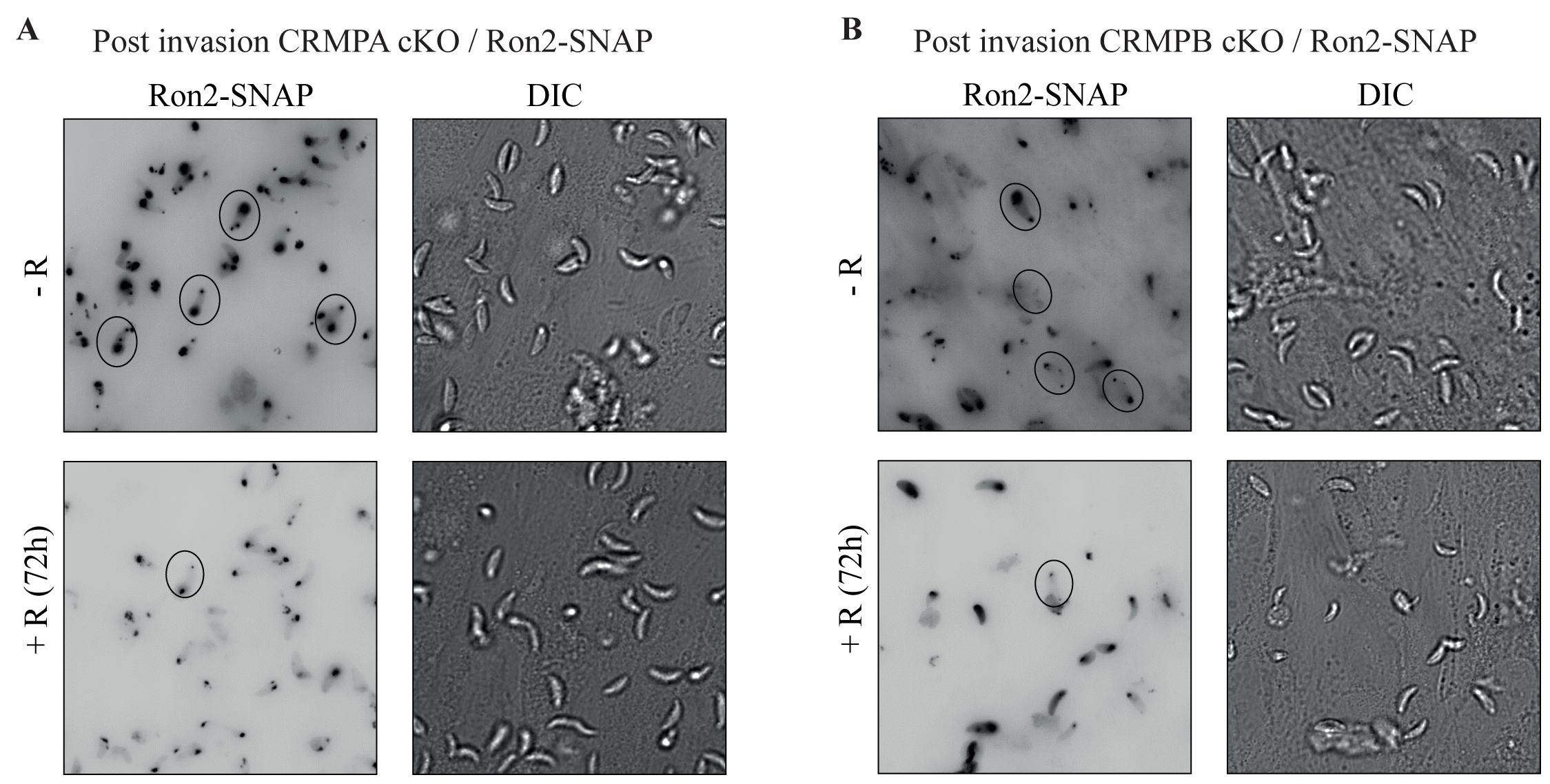

Supplement: S6 Fig — In the cKO parasites of CRMPB and CRMPA, RON2 was endogenously tagged with SNAP. Shown are maximum projections after 20 min of invasion (72 h post-rapamycin induction); invaded parasites are circled. Both invaded parasites in the plus rapamycin group represent the only tachyzoite with a clear “invaded” RON2 staining pattern; minus rapamycin are representative stacks. (TIF) [file pbio.3001937.s014.tif]

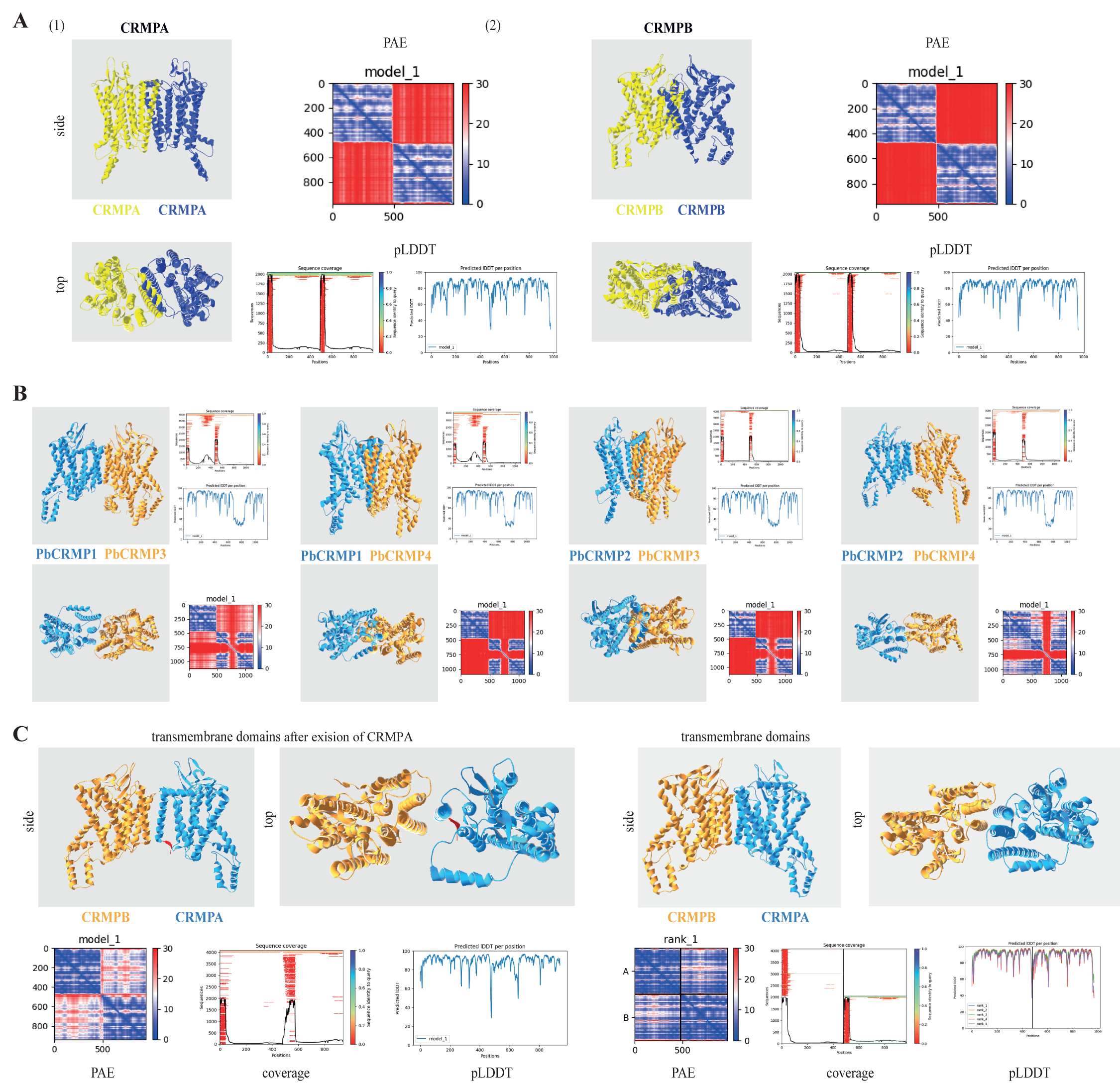

Supplement: S7 Fig — (A) Homodimer modeling for (1) CRMPA and (2) CRMPB result in overlay of proteins. The PAE scores show good relative prediction for single chains but indicate that both chains do not interact, despite the high IDDT values. Sequence alignment blots indicate the number of sequences that could be aligned to each position. (B) Multimer heterodimer prediction of PbCRMP1/3, PbCRMP1/4, PbCRMP2/3, and PbCRMP2/4. CRMPA clade members are colored light blue; CRMPB clade members are colored orange. PAE scores indicate that PbCRMP1/3 and PbCRMP2/4 do interact and that PbCRMP1/4 and PbCRMP2/3 do not interact. PbCRMP3 and PbCRMP4 are predicted in two fragments with an unstructured part (indicated by the low local pLDDT score). (C) Prediction of heterodimer formation of CRMPA and CRMPB transmembrane domains after induction of RH-CRMPA-3HAfloxed resulting in loss of the C-terminus and the last 2 transmembrane domains. New C-terminus is indicated in red. Notice the change in PAE score compared to full-length TMs shown on the right. Input sequences are in S2 Table, Structure B-H in S1 File. (TIF) [file pbio.3001937.s015.tif]
