# Supplementary material for: A central CRMP complex essential for invasion in Toxoplasma gondii
Source: PLoS Biol. 2023 Jan 5;21(1):e3001937. doi: 10.1371/journal.pbio.3001937 (PMC9815656; doi:10.1371/journal.pbio.3001937)

A S2E Fig PCR raw image

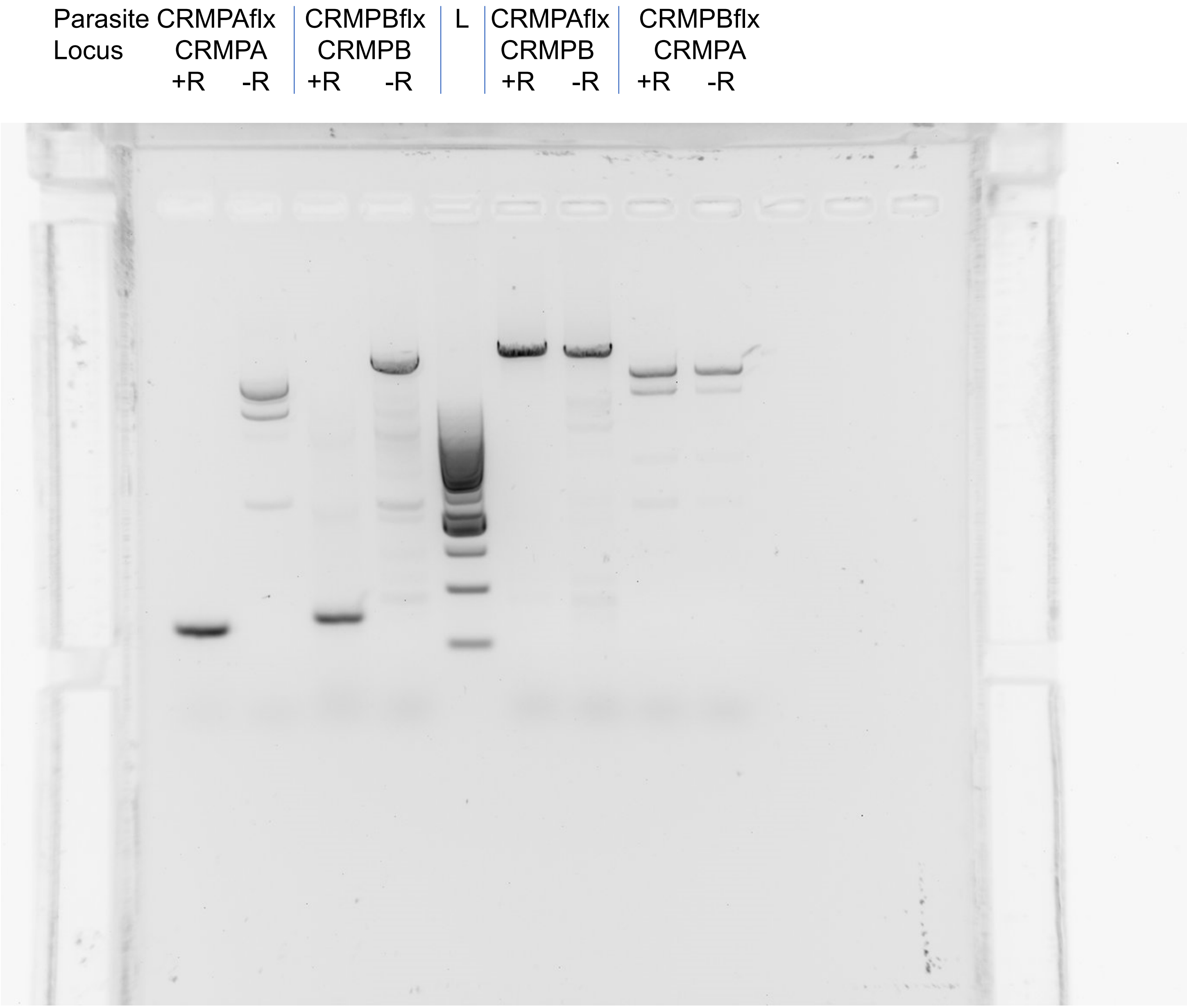

B S3A Fig micronemal secretion Gel 1

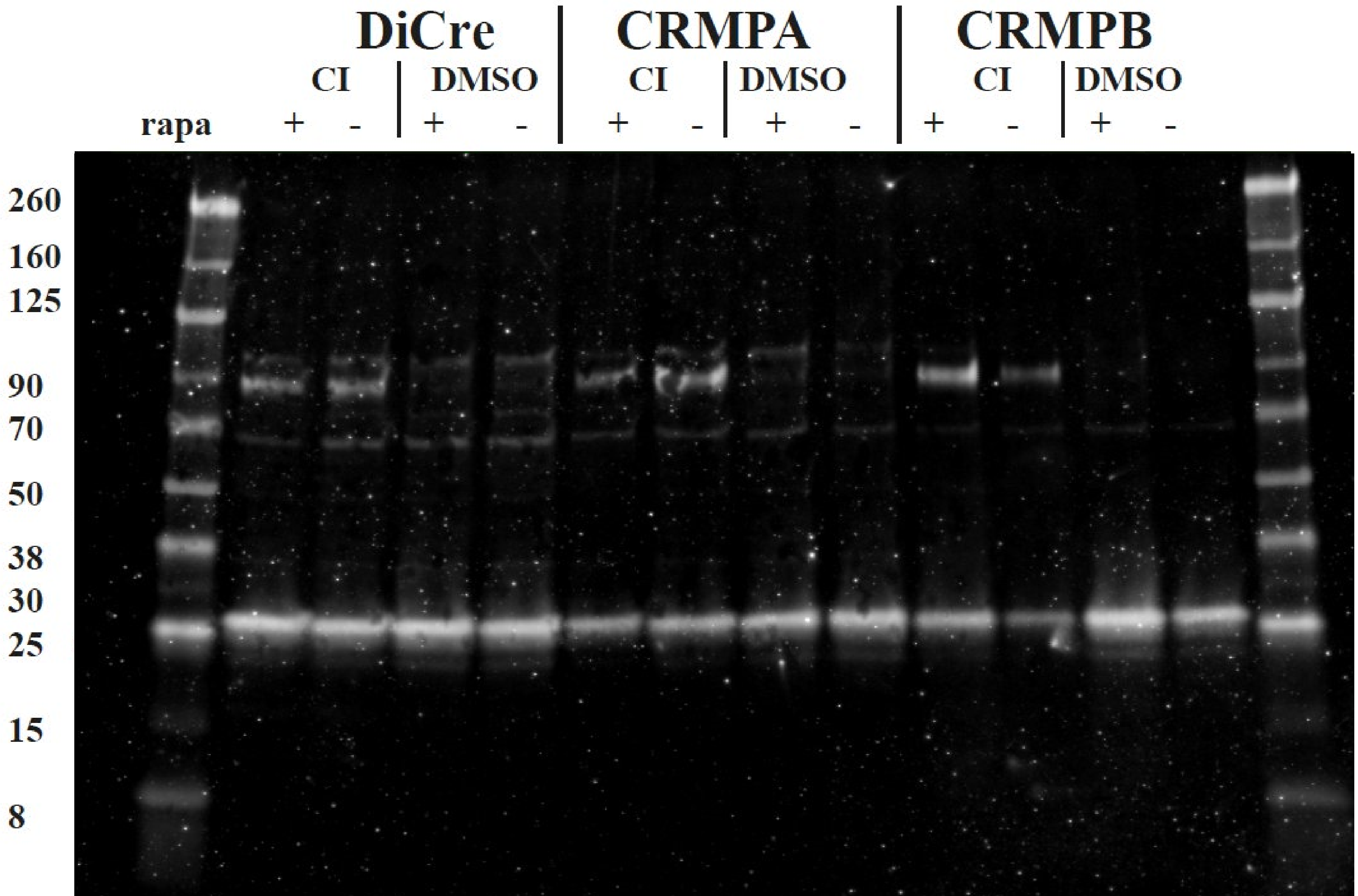

C S3A Fig micronemal secretion Gel 2

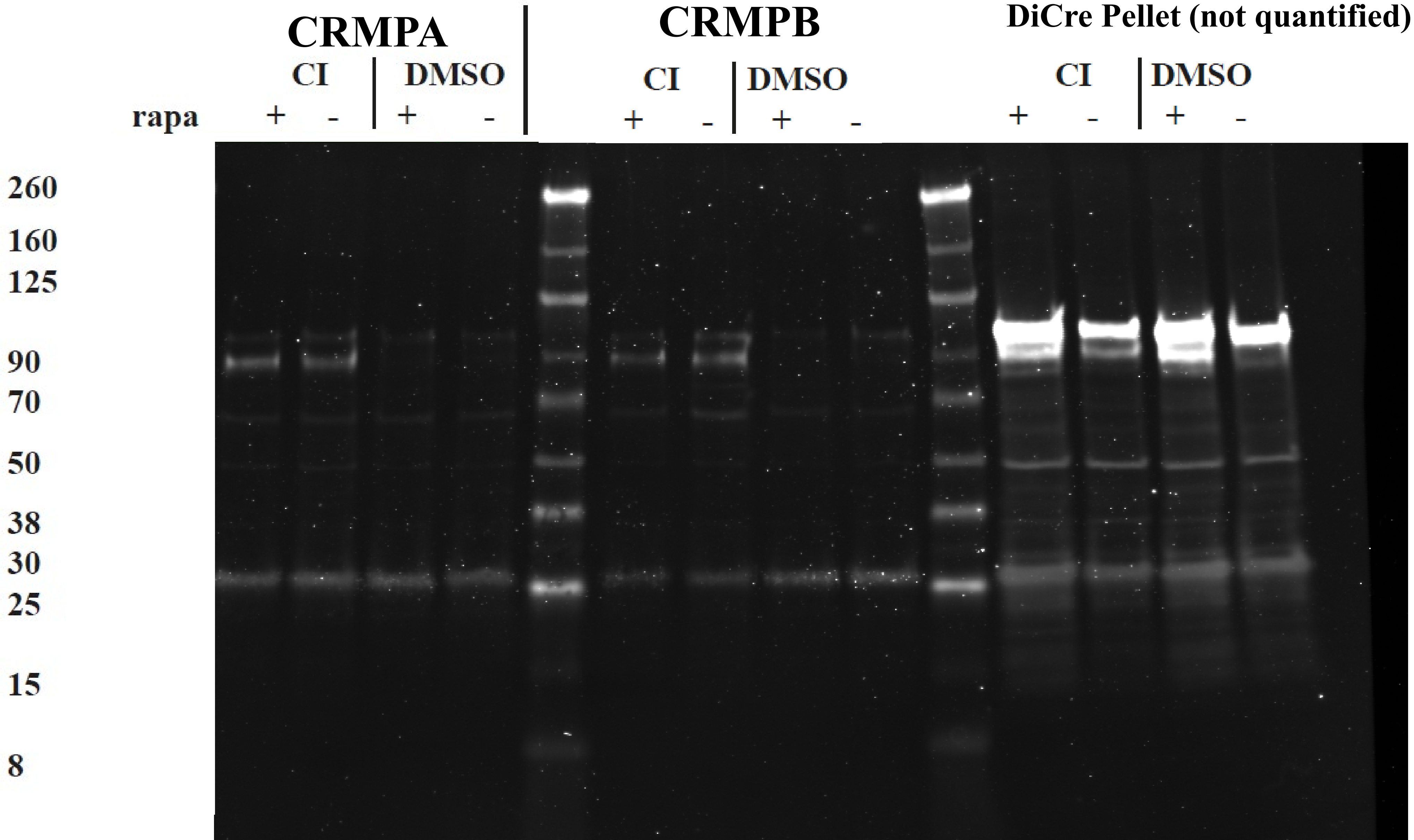

D S3A Fig micronemal secretion Gel 3

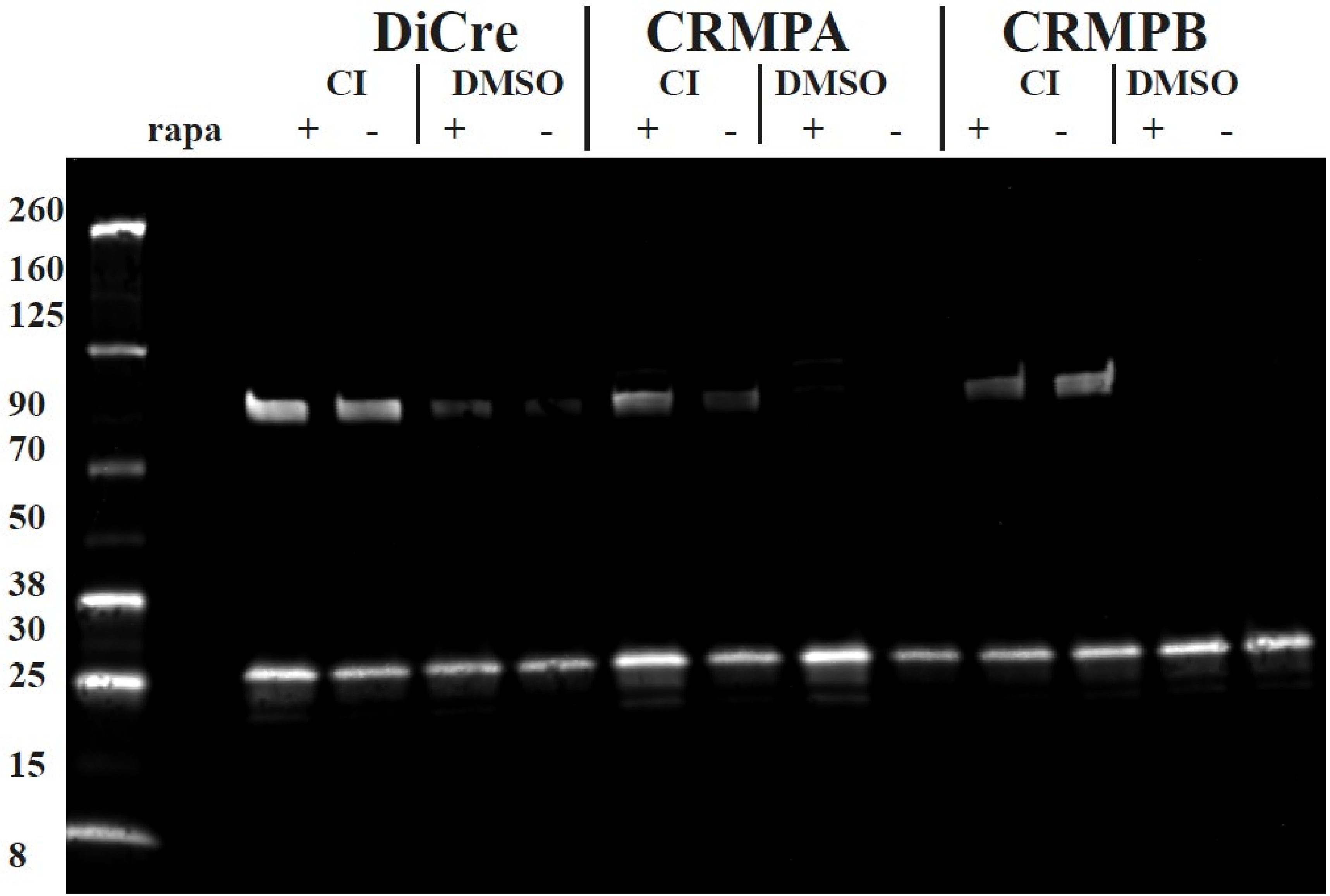

Supplement: S1 Raw Images — (A) Raw image of DNA agarose gel shown in S2E Fig. On the right side, the negative control (genotyping the non-floxed locus) is shown (data not included in S2E Fig). (B-D) Raw images of western blots used for the quantification of micronemal secretion in S3A Fig. MIC2 (upper bands) and GRA1 (lower band) are probed with the same secondary antibody 72 h post-rapamycin treatment. For details, see Methods section. (PDF) [file pbio.3001937.s016.pdf]
