# Supplementary material for: A central CRMP complex essential for invasion in Toxoplasma gondii
Source: PLoS Biol. 2023 Jan 5;21(1):e3001937. doi: 10.1371/journal.pbio.3001937 (PMC9815656; doi:10.1371/journal.pbio.3001937)
Supplement: Attachment — Submitted filename: Response to reviewers-final.docx [file pbio.3001937.s018.docx]

**Response to reviewers**

We would like to thank the reviewers for their time and constructive comments that helped to improve our manuscript.

In our revision we provide additional data to address the reviewers and Academic Editor’s comments. Briefly, we performed many additional experiments, such as the generation of an additional TurboID dataset to demonstrate interactions of CRMPs with MIC15, microneme secretion assays and measured rhoptry secretion via Stat-6 phosphorylation.

We also edited the manuscript and clarified the reviewers concerns. All line numbers mentioned correspond to the document with track changes.

We included additional references into the study about recently published work about MIC15 (lines 604-605) and the competing work about the CRMPs (lines 644-645) (the latter is accepted so the reference can potentially be updated in the next days).

Please find our detailed response below (in blue):

Reviewer #1: In this manuscript, the authors characterized a novel protein complex in Toxoplasma parasites for rhoptry release, further impairing parasite invasion. Two cysteine repeat modular proteins (CRMPs), named CRMPA and CRMPB, are apicomplexan-specific members. The authors used the Di-Cre system to delete individual CRMP proteins and found that both are essential for parasite invasion. Further, the authors used pull-down and BioID assays to determine the interacting partners with both CRMPs. Two proteins, Mic15 and TSP1, were enriched as the top candidates interacting with both CRMPs. The manuscript took multiple strategies to discover and validate protein-protein interactions and the subcellular localization of the proteins of interest, which enhances the rigor of this work. In addition, the generation of multiple knockouts for the components within this essential protein complex strengthens the conclusion. Overall, this manuscript studied a fundamental question of
how the apicomplexan parasites shed their invasion factors for their infection. The authors provided compelling data from microscopy and phenotypic assays for proving their hypothesis. I have a few comments below.

We thank the reviewer for the very positive and constructive feedback. We hope that we addressed all comments in our revision:

1. The authors identified a novel protein complex comprising CRMPA, CRMPB, Mic15, and Tsp1. I wonder if Mic15 and Tsp1 interact with CRMPA or CPMPB directly or they associate with CRMPA via CPMPB and vice versa? A biotinylation assay in the strains lacking CRMPA or CRMPB may answer this question.

We appreciate this suggestion and performed this experiment. We generated a third TurboID dataset, using two parasite lines with the other CRMPs floxed and repeated the 30 min extracellular biotinylation 72 h after induction with rapamycin (now dataset 4 TurboID see supplementary Table 1). As the – rapamycin control now also provides data for both CRMPA-TurboID and CRMPB-TurboID, we also updated this accordingly in Figure 4 (see lines 333-334, 347-348).

To discuss these new data, we introduced a new paragraph after the data for Mic15 and TSP1 (now lines 409-431), and modified Figure 6 (A-D) accordingly (lines 434-437). The data suggests that Mic15 interacts directly with CRMPA and TSP1 interacts with CRMPB. The data do not appear entirely “mirrored”, and we expect this due to residual CRMPA protein levels after induction with rapamycin (the N-terminus and the remaining transmembrane domains), whereas induction of CRMPB results in reduced CRMPA as previously shown in Figure 3 D and E. We modeled the TMs of CRMPA after excision of the last 2 TMs together with CRMPB (Figure S7 C), suggesting that they might still interact. The method section has been updated accordingly (lines 709-721).

2. Does the depletion of CRMPA or CRMPB affect the secretion of microneme proteins? It would be a good control for distinguishing the roles of microneme and rhoptry release in parasite invasion.

Since we observed no difference in gliding motility, which depends on microneme secretion, we initially felt that a separate secretion assay is unnecessary. However, the reviewer is correct that we might have missed minor differences and therefore performed a micronemal secretion assay, as suggested. We did not detect an observable defect on secretion, supporting our hypothesis. The quantification is now shown in new Figure S3A and we discussed the data briefly in lines (281-282).

3. The authors mentioned several previously reported determinants for rhoptry release. It would be helpful to dissect if the depletion of CRMP complexes will not affect other pathways.

We performed a colocalization with Ama1 and Mic8 in the presence and absence of CRMPB (now Figure S3 B and D). We chose not to do this for Nd6 and Nd9 as we knew that the Lebrun lab, who recently identified these proteins, was already doing those experiments.

We also screened all our TurboID data for all previously known rhoptry secretion determinants and did not observe any enrichment (now line 428-431).

Currently there is no signaling pathway described for rhoptry secretion which could be tested to see which factors act up- or downstream. Therefore, we agree that our current model is hypothetical.

4. Although the authors predicted the structures of CRMPs using the latest algorithm to build the 3-D model of the CRMP complexes, I feel the authors may need to keep cautious to use this model for speculation and prediction. It would be great to move this part from the results to the discussion section.

We agree that experimental verification of the models would be great, but this is beyond our expertise and scope of this manuscript. We feel that there is great promise of the suggested interaction via the transmembrane domains, which is supported in all combinations (lack of homo-dimerization of CRMPA or CRMPB, same for PbCRMPs. Additionally this is now supported with the new TurboID dataset, suggesting residual interaction between CRMPA and CRMPB after excision of CRMPA (the last two transmembrane domains and the C-terminus)(Figure S7 C).

Therefore, we agree that the current model will require further experimental validation either from in vitro expression of both TMs or a cryoEM structure in the future.

According to the reviewers suggestions, we decided to move this paragraph to the discussions section (now lines 570-593).

Reviewer #2: This manuscript present new findings on two Cysteine Repeat Modular Proteins in T. gondii.  CRMPA and CRMPB are homologous of proteins in Plasmodium, where they have been shown to be important in the invasion by sporozoites, but interestingly not merozoites. These orthologs were also predicted by be essential in a genome wide CRISPR screen in T. gondii.  Hence the investigators take the rather predictable approach of deleting them using an inducible DiCre system to test their function.  Not unsurprisingly, they are found to be important.  The key new finding is that these proteins appear to be necessary for rhoptry secretion.  Additionally, there is evidence that they interact with microneme proteins in a complex, which is needed for invasion.  What is less clear is why the distribution of the proteins (the secretory system in general) does not match the putative function (apical complex).  It is also unclear why the authors propose a complex model for the
extracellular domains of CMRPA/CRMPB that is not well supported by data, while more direct alternatives are not acknowledged.

We thank the reviewer for the critical comments that helped us to improve the manuscript. While we agree that our approach might be seen as “predictable”, we believe that the importance of this work is underlined by the fact that concurring studies have been performed in (at least) 2 other laboratories, demonstrating that there is huge interest in the identification of factors required for rhoptry secretion, a key step of the invasion pathway. We also believe that our data not only show that these proteins are required for rhoptry secretion, but that they form a novel complex AND we identified additional interaction partners of this complex.

We are unsure, why the reviewer thinks that the distribution of the proteins doesn’t match the well demonstrated function in rhoptry secretion. We now provide additional data for this function, in case there are lingering doubts on the reviewers mind regarding these findings.

As mentioned above, we moved the complex interaction model now into the discussion section. We do think that the extent of our data allows us to formulate a working model that can be followed up in future experiments and either be validated or invalidated.

Specific comments

1)      The abstract is unnecessary complicated by negative comments about prior work on individual microneme proteins.  Not expectantly, many microneme proteins have overlapping or redundant roles and hence knockouts often do not reveal strong or "essential phenotypes".  However, this is not the point of the present manuscript, which rather focuses on a new complex important for rhoptry secretion and invasion.  I would suggest focusing on the positive data for the new findings rather than disparaging previous work for perceived deficiencies that are entirely consistent with the biology.

We never intended to discredit previous work, and some of this work has been done by our laboratory. We are somewhat surprised that it was taken as such and now mention the “functional redundancies” as suggested by the reviewer (now lines 28-29).

2)      The localization data for CRMP1 and CRMPB is hard to reconcile with the proposed function in ROP secretion.  The CRMP proteins appear to be widely distributed in the secretory pathway, but only transiently or weakly associated with the apical tip.  The use of an N terminal tag is used to show that CRMPs are secreted to the surface, but these experiments are complicated by the fact that parasites are imaged after invasion, leaving a lot of time for redistribution (Fig 2C).  Additionally, the parasites being imaged appear to express YFP, and hence it is not clear that the detection of GFP added prior to invasion is not simply due to cross reaction. Many antibodies to GFP cross-react to YFP and it is not clear what is the specificity of the reagents used here? Regardless, it would more informative to visualize the extracellular tag in parasites that are free of host cells, for example when stimulated to secrete micronemes and when the conoid is extended.  Is there evidence
for external display of the protein at the apical end in such parasites?

We agree with the reviewer that most CRMP proteins can be found within the secretory pathway, while the expected place of action would be at the surface of the parasite, post secretion. However, in case CRMPs are triggering a signaling cascade, a defined apical location might not be necessarily required. Other secreted proteins are known to have a similar ratio of active protein vs total stored protein, as for example MIC2 or MIC8 (another protein required for rhoptry secretion). It is very challenging to formally show that the localization of only a subset of the protein is the only place its activity is essential.

The anti-GFP antibody we used is a polyclonal anti-GFP antibody that detects all forms of GFP including the sYFP2 used, we updated the methods accordingly (lines 802-803).

The reviewer suggested a very interesting experiment, which we actually attempted to perform prior to submission of the manuscript. Briefly, we tried to image extracellular parasites that have been stimulated for microneme secretion. We tried to use parasites with N-terminal sYFP2-tagged CRMPs stained with anti-GFP and also N-terminally Halo-tagged CRMPs labeled with a non-membrane permeable Halo dye. Unfortunately, in both cases signal over noise was too weak to reach a definite conclusion.

Therefore, we focused on the post invasion experiment shown in Figure 2C, as well as those shown in Figure 3D and E. Here, the parasites express both N-terminally sYFP2-tagged CRMPB as well as C-terminally sYFP2-tagged CRMPA. While the experiment does not formally show which of those YFPs is detected on the host cell surface post invasion, it does demonstrate delivery of CRMPs to the surface of the parasite.

The supplementary movie 2 of Halo labeled parasites suggests some apical display and maybe a transient labeling of the moving junction during invasion (see Supplementary movie 2).

In summary, we do think that our data convincingly show delivery of these proteins to the surface.

3)      Although the authors favor a view that CRMPs are needed for ROP secretion, their primary localization does not match this function but rather appears to be at the posterior end (after invasion).  As well, they appear to be broadly distributed in the secretory pathway.  It is unclear why they would have a role in triggering ROP secretion, given this broad distribution.  It suggests that the phenotype of failed ROP secretion is indirect, perhaps due to another protein that fails to traffic correctly to the apical end?   MIC15 and TSP1 appear to be good candidates for this function since they lose apical localization on knockout of CRMPB. It would be informative to test if evacuoles are also disrupted by knockout of MIC15 and/or TSP1. The authors do not adopt this conclusion but rather favor a more complicated, and less well supported, conclusion that the extracellular domains of CRMPs are involved in signaling.  If that were the case, the truncations that lack the
extracellular domain might be informative. In the absence of such data, I do not see a compelling argument for rejecting the more direct conclusion that CRMPA/CRMPB are part of the secretory pathway and are required for trafficking of MIC15 and TSP1, which are in turn required for ROP secretion.

All four proteins, Mic15 and TSP1, as well as the CRMPs colocalize perfectly (see Figure 5 B). Although we understand somewhat the view of the reviewer, as mentioned above, CRMPs are detected on the surface and given their potential role as signaling factors do not necessarily need to be concentrated at the tip of the parasite. In fact, the microneme proteins MIC8 (Kessler et al., 2008) and CLAMP (Sidik et al., 2016) show a similar localization and behavior. In both cases the respective protein is localized in the micronemes and (in case of CLAMP) it has been shown that:

“Following incubation with host cells, formation of CLAMP foci could be detected at the **posterior** of most parasites (Figure S5A). We also observed active formation of such foci during invasion (arrowheads, Figure 6E, & Movie S2). **This relocalization of CLAMP is similar to what has been observed for other membrane-tethered micronemal proteins (Carruthers and Sibley, 1999; Garcia-Reguet et al., 2000).”**

In order to provide additional evidence regarding the crucial function in rhoptry secretion, we now used the well accepted Stat6-phosphorylation assay (now Figure 5 D). This data demonstrate that each of the four proteins is required for rhoptry secretion. As for the effect on invasion (Figure 3C, Figure 5G) the disruption is more pronounced for the CRMPs than seen for Mic15 and TSP1. This is mentioned in lines 386-387, 405-406.

As mentioned above, based on the suggestion by reviewer 1, we now also generated a new TurboID dataset comparing two parasite lines with one CRMP tagged with TurboID and the other CRMP floxed and repeated the 30 min extracellular biotinylation 72 h after induction with rapamycin (see our response above to reviewer 1, comment 1). These data demonstrate that Mic15 interacts directly with CRMPA and TSP1 interacts with CRMPB.

In conclusion Mic15 and TSP1 seem to interact with one CRMP each, which in turn interact with each other. It is hard to decipher between effects of trafficking (which seem only partial and only for CRMPB) and complex breakdown, but the easiest interpretation is that the CRMP complex requires the presence of all four proteins to trigger rhoptry secretion.

4)      The material presented in Figure S7 is highly speculative and premature.  I agree that domain predictions shown in Figure 6A are interetsing, but they only comprise a small portion of the mature CMPR proteins and hence cannot be used to infer their overall structure much less function.  I would suggest removing these speculative sections for future studies that might provide greater mechanistic support for the claims.

See our response above to a similar comment from reviewer 1, comment 4. We now removed all predicted structures except the transmembrane domains from the Figures and moved the chapter into the discussion, to clarify that this is still a prediction. We also rephrased this part to highlight its predictive nature (now lines 570-593).

5)      It is unclear why the authors choose to name the Tg orthologs CRMPA and CRMPB when in other apicomplexans they are numbered.  This seems likely to lead to confusion.

We did this in agreement with two other labs working on the same proteins, demonstrating the huge interest in these proteins. We therefore defined the A clade with CRMPA in coccidia and CRMP1 and CRMP2 in piroplasmida, as well as the B clade with CRMPB in coccidia and CRMP3 and CRMP4 in piroplasmida. Labeling them CRMP1 and CRMB2 would cause confusion regarding their homologues in Plasmodium species. We also explain this in lines 129-131.

Reviewer #3: Singer et al. provide compelling evidence for a novel invasion complex in Toxoplasma gondii that may answer the longstanding question of how rhoptry secretion is triggered. Prior to publication, I recommend that the reviewers address the following concerns:

We thank the reviewer for the overall positive feedback. We hope that all concerns are addressed accordingly in our revision.

Major Comments
1.      The claim that TSP1 and MIC15 function in a complex with CRMPA and CRMPB requires further investigation to be convincing. They appear less important for plaque formation and invasion than CRMPA and CRMPB, and their expression and localization are not impacted by loss of CRMPB (unlike CRMPA). E-vacuole formation (rhoptry secretion) was not tested for TSP1 and MIC15 either. These data suggest they could be in a separate, nearby complex. One suggestion to strengthen this claim could be to do the reciprocal co-IP with detection of binding partners by immunoblotting (i.e. pull down TSP1 and MIC15 separately and immunoblot for CRMPA and CRMPB). Otherwise, in vitro binding experiments could be performed with recombinant proteins.

See our response above to reviewer 1, comment 1. We performed two new experiments which address these concerns, which are now shown in Figure 5 D (rhoptry secretion measurement for all four proteins using the Stat-6 phosphorylation assay) and a new mass spectrometry experiment using RH-CRMPA-3HA^floxed^ RH-CRMPB-TurboID and RH-CRMPB-3HA^floxed^ RH-CRMPA-TurboID + / - rapamycin induction 72 h prior to biotinylation (now dataset 4 TurboID see supplementary Table 1) and Figure 6 A-D (386-387, 405-406, 409-431, 434-437).

The new rhoptry secretion measurement shows a very similar results as the invasion assay (Figure 3C and 5G), with the CRMPs having a slightly stronger phenotype that MIC15 and TSP1.

Minor comments
1.      Invasion doesn't need to be capitalized in title.

done
2.      Abstract: "Here we demonstrate that both proteins form a complex that contains additional micronemal proteins". This statement is ambiguous as it could be interpreted that CRMPA and CRMPB are micronemal proteins, which it appears that they are not.

Yes, we agree, while they share some features with micronemes, they do not associate with microtubule associated micronemes (Figure 3 B-D). To avoid confusion, we now rephrased line 33-35: “*Here we demonstrate that both proteins form a complex that contains the additional proteins Mic15 and the thrombospondin type 1 domain-containing protein (TSP1).”*

3.      Intro: "at least two addition" to "at least two additional".

Done, now line 113

4.      Fig 1A: tree appears cut off on left side, the outgroup with 6 accessions (e.g. DICDI Q86I19) looks disconnected. Indicate color coding in legend, "colored by apicomplexan species".

Done, legend now in line 155
5.      Fig 1B: aa numbers are unnecessarily small and illegible.

corrected
6.      Consider using US/UK period for decimal points instead of comma for this journal (e.g. "phenotypic score of -3.95 and -3.64 respectively".

done
7.      Explain the general functions of Kringle, GCC2, and GCC3 domains in the intro, results or discussion.

Thanks for pointing this out. We initially kept both within the manuscript as these are the current names on Toxodb. We will aim to change both to CRMPA and CRMPB, and then the reference to the mislabeling with GCC2 and GCC3 domain containing protein can be removed from the paper. We changed the abstract accordingly, lines 30-32 now read: *“Cysteine Repeat Modular Proteins (CRMPs) are a family of apicomplexan specific proteins. In Toxoplasma gondii, two CRMPs are present in the genome, CRMPA (TGGT1_261080) and CRMPB (TGGT1_292020).”*

In the results sections we also changed this part, now lines 147-151: *“Both proteins contain several cysteine-rich domains and 9 transmembrane domains. CRMPA also contains a Kringle domain that has been shown to be involved in protein-protein interaction {Patthy, 1984 #89} (Figure 1 B).”*

8.      Results: "To determine if these proteins are delivered to the plasma membrane," to "To determine if CRMPB is delivered to the plasma membrane," . sYRP2-CRMPB but not sYFP2-CRMPA was tested in Fig 2C.

Corrected, now line 205
9.      Fig 2: Results beg the question, is CRMPA and CRMPB on the surface of extracellular parasites or only displayed following contact with host cells or microneme secretion?

See also our response to reviewer 2, comment 2. We agree. The main pool of the CRMPs remains intracellular also during invasion. We tried to address this in CI stimulated extracellular parasites using anti-GFP antibody (detecting YFP) detecting the N-terminally sYFP2-tagged CRMPB in unpermeabilized parasites as well as staining an N-terminal Halo-tagged CRMP with a non-membrane permeable dye but could not generate sufficient signal to noise compared to the DiCre control (this is why we then performed the post invasion assay in Figure 2). We speculate that CRMP secretion is linked/similar to microneme secretion.

10.     Fig 3A: since plaque area wasn't quantified, discuss apparent growth defect caused by floxing CRMPA (in absence of rapamycin as compared to the parental line). Does floxing CRMPA reduce basal levels or cause alternative splicing?

We realized that we badly chose a non-representative plaque assay for CRMPA, and did replace the one shown in Figure 3A. As suggested, we no discuss this, lines 274-276 now reads: “*For both CRMPs, insertion of the LoxP site did not result in any observable fitness loss or misslocalization (Figure 3 A, Figure 2 A, Figure S4 B).”* We did not check for alternative splicing.
11.     Results (Fig 3C): state whether CRMPA and CRMPB were confirmed to be knocked out in the vacuoles counted for replication.

We updated the method section, lines 839-842 now reads: *”After 24 h, cells were fixed and stained with α-Gap45 and α-HA. In the +R sample, no intracellular parasites were observed that expressed detectable HA. Relative parasite number within vacuoles was counted for three biological replica.”*

12.     Fig 4A: "massspec" to "mass spec"

done
13.     Results: "While deletion of TGG1_285000" to "While deletion of TGGT1_285000"

done, now line 398

14.     Results: "Similarly, deletion of tetratricopeptide protein (TGG1_247000)" to "Similarly, deletion of tetratricopeptide proteins (TGGT1_247000)"

done, now line 401
