# Supplementary material for: A central CRMP complex essential for invasion in Toxoplasma gondii
Source: PLoS Biol. 2023 Jan 5;21(1):e3001937. doi: 10.1371/journal.pbio.3001937 (PMC9815656; doi:10.1371/journal.pbio.3001937)
Supplement: Attachment — Submitted filename: Response to reviewers.docx [file pbio.3001937.s019.docx]

Response to reviewers

Reviewer #1: The authors did the experiments suggested by the reviewers and also incorporated the results, explanations, and discussions into the text. I do not have further comments. Congratulations to all authors!

Thank you for your time and productive discussion.


Reviewer #2: I would like to thank the authors for their thorough replies and for addition of new data to support their conclusions. I think many of the points claimed here are well supported including: 1) CRMPA and CRMPB interact with each other, 2) CMRPs are widely distributed in the secretory pathway and partially overlap with MIC proteins, although they are not themselves contained in micronemes, 3) CRMPA primary interacts with MIC15 while CRMPB primarily interacts with TSP1, 3) All 4 proteins form a complex that is necessary for ROP secretion. The one claim that I am not convinced by is the conclusion that CRMP1 and CRMPB are on the surface and that they might require this location for function. The data presented in Figure 2 is by the authors own estimation "suggestive" (lines 194, 200) and even if it does indicate that a small portion of CRMPA and CRMPB reach the surface it does not show that this location is critical to their function. Additionally, the loss of CRMPs results in mislocalization of MIC15 and TSP1 so that they are now retained in the ER. The resulting mislocalization could explain the phenotypes of both the CRMP conditional knockdowns and the MIC15 and TSP1 knockdowns. As the current data cannot distinguish between these models, they need to soften the claims about CRMPs functioning at the surface and add this alternative explanation to the discussion.

Thank you for your time and productive discussion.

We now specify the linked phenotypes being explained by complex disruption in absence of one member as well as escorter function of CRMPs for MIC15 and TSP1. We added this in lines 514-518 in the discussion:

*“Thus, absence of CRMPB will result in mistrafficking of TSP1 and CRMPA (Figure 3 E) which will subsequently affect MIC15. Absence of CRMPA will mainly affect MIC15. Since the strength of the phenotype of CRMPA and CRMPB are identical and more severe than MIC15 and TSP1 it is likely that in addition to the proposed function at the surface, CRMPA and CRMPB act as escorter for other components of the complex.”*

*Additionally we now discuss the related publication (doi:[10.15252/embj.2022111158](https://www.embopress.org/doi/full/10.15252/embj.2022111158)) in more detail. Together with our statement in line 211:*

“We have however no indication which of the observed localizations is functional for invasion.”

*This now is discussed in respect of their more precise localization data. (line 580-584)*

“Here, the same four members of the CRMP complex were identified, shown to be secreted and the same phenotype using the mAID system was observed. The CRMPs also colocalize with the Nd complex at the extruded conoid. Colocalization and the identical phenotype and absence of proximity labeling suggests they might be interlinked by an unknown mediator.”

Suprisingly, the mAID system they used caused functional block in invasion after 48h coinciding with loss of apical secreted localization, while intracellular localization was only slightly decreased. This strongly suggests that secretion is essential for function of CRMPs , either directly or indirectly as escorters.


Reviewer #3: Singer et al. have adequately addressed all of my concerns in the revised version of this manuscript. Upon review, no additional concerns were noted in the revised manuscript.

Thank you for your time and productive discussion.
